# Pathomechanisms of HIV-Associated Cerebral Small Vessel Disease: A Comprehensive Clinical and Neuroimaging Protocol and Analysis Pipeline

**DOI:** 10.3389/fneur.2020.595463

**Published:** 2020-12-15

**Authors:** Kyle D. Murray, Meera V. Singh, Yuchuan Zhuang, Md Nasir Uddin, Xing Qiu, Miriam T. Weber, Madalina E. Tivarus, Henry Z. Wang, Bogachan Sahin, Jianhui Zhong, Sanjay B. Maggirwar, Giovanni Schifitto

**Affiliations:** ^1^Department of Physics and Astronomy, University of Rochester, Rochester, NY, United States; ^2^Department of Microbiology and Immunology, University of Rochester, Rochester, NY, United States; ^3^Department of Electrical and Computer Engineering, University of Rochester, Rochester, NY, United States; ^4^Department of Neurology, University of Rochester, Rochester, NY, United States; ^5^Department of Biostatistics, University of Rochester, Rochester, NY, United States; ^6^Department of Imaging Sciences, University of Rochester, Rochester, NY, United States; ^7^Department of Neuroscience, University of Rochester, Rochester, NY, United States; ^8^Department of Microbiology, Immunology, and Tropical Medicine, The George Washington University, Washington, DC, United States

**Keywords:** HIV, cerebral small vessel disease, neuroinflammation, MRI, vascular health, image processing pipeline

## Abstract

**Rationale:** We provide an in-depth description of a comprehensive clinical, immunological, and neuroimaging study that includes a full image processing pipeline. This approach, although implemented in HIV infected individuals, can be used in the general population to assess cerebrovascular health.

**Aims:** In this longitudinal study, we seek to determine the effects of neuroinflammation due to HIV-1 infection on the pathomechanisms of cerebral small vessel disease (CSVD). The study focuses on the interaction of activated platelets, pro-inflammatory monocytes and endothelial cells and their impact on the neurovascular unit. The effects on the neurovascular unit are evaluated by a novel combination of imaging biomarkers.

**Sample Size:** We will enroll 110 HIV-infected individuals on stable combination anti-retroviral therapy for at least three months and an equal number of age-matched controls. We anticipate a drop-out rate of 20%.

**Methods and Design:** Subjects are followed for three years and evaluated by flow cytometric analysis of whole blood (to measure platelet activation, platelet monocyte complexes, and markers of monocyte activation), neuropsychological testing, and brain MRI at the baseline, 18- and 36-month time points. MRI imaging follows the recommended clinical small vessel imaging standards and adds several advanced sequences to obtain quantitative assessments of brain tissues including white matter microstructure, tissue susceptibility, and blood perfusion.

**Discussion:** The study provides further understanding of the underlying mechanisms of CSVD in chronic inflammatory disorders such as HIV infection. The longitudinal study design and comprehensive approach allows the investigation of quantitative changes in imaging metrics and their impact on cognitive performance.

## 1. Introduction

Cerebral small vessel disease (CSVD) is a common neurodegenerative disease associated with aging and other neurological diseases that is diagnosed by brain imaging. CSVD affects small penetrating arteries, arterioles, capillaries, and venules (1) and is clinically diagnosed by presence of brain atrophy, white matter hyperintensities (WMHs), enlarged perivascular spaces, cerebral microbleeds, and lacunar infarcts. These are detected by T1-weighted (T1w), fluid attenuated inversion recovery (FLAIR), T2-weighted (T2w), T2*-weighted (T2*), and diffusion MRI (dMRI) (2). Additional neuroimaging techniques to study CSVD in more detail have been proposed and include (3) cutting-edge research sequences that can be used to assess altered microcirculation and blood brain barrier (BBB) dysfunction indirectly by measuring cerebrovascular reactivity (CVR), cerebral blood flow (CBF), white matter microstructure, and tissue susceptibility (4). As further detailed below, in this study we used the following advanced research sequences to obtain quantitative tissue metrics throughout the brain: multi-shell dMRI, resting-state functional MRI (rs-fMRI), multiple-delay pseudo-continuous arterial spin labeling (pcASL), and quantitative susceptibility mapping (QSM).

Older adults living with human immunodeficiency virus (HIV) infection are at increased risk of developing cerebrovascular disease in terms of both large and small vessel disease (5–7). Relatively limited attention has been devoted to HIV-associated CSVD so far, despite its known long-term effects on cognitive function in the general population. In HIV-infection, CSVD could be the result of multiple mechanisms (8–10). In addition to the known atherosclerosis mechanisms affecting the older population, older HIV+ individuals are also exposed to HIV proteins, host immune activation products, and combination antiretroviral therapy (cART), which can affect the neurovascular unit (endothelial cells, pericytes, astrocytes, neurons, and microglia). Interestingly, CSVD has been associated with markers secreted by myeloid cells (11, 12).

Aberrant platelet activation during HIV infection causes an increase in platelet-monocyte complexes (PMCs) that drives monocyte maturation from CD14+/CD16- to the proinflammatory CD14(low)/CD16+ phenotype (13, 14). Reduction in the numbers of CD14+/CD16- monocytes are associated with proatherosclerosis changes (15, 16), BBB permeability (17, 18), and aging (19, 20). Based on these observations, we hypothesize that pro-inflammatory monocytes lead to an altered integrity of the BBB and neurovascular unit. Therefore, in this study we investigate mechanisms by which activated platelets interacting with monocytes and endothelial cells contribute to CSVD, in particular via processes that alter vascular permeability and reactivity which lead to altered microstructural integrity. We employ standard MRI sequences used in clinical practice to qualitatively ascertain the presence of CSVD and four advanced MRI sequences to quantitatively asses white matter structural integrity, vascular reactivity, CBF, and iron distribution. We also incorporate novel markers of immune activation and cognitive testing.

## 2. Methods and Analysis

### 2.1. Study Design

A total of 110 HIV-infected (HIV+) men and women and 110 HIV-uninfected (HIV-) age and sex matched controls will be recruited using recruitment materials approved by the Institutional Research Subjects Review Board (RSRB). All participants provide written informed consent according to the RSRB approved protocol prior to any evaluation. Blood samples are acquired from adults after written informed consent carried out in accordance with the Declaration of Helsinki. Study participants are followed for 3 years as changes in CSVD are expected to occur within 3 years of follow-up.

The inclusion and exclusion criteria are shown in Table 1. Special effort will be made to enroll controls that are demographically similar to the HIV+ subjects. Both men and women are invited to participate in the study as there are no scientific bases for sex restrictions. However, given the sex distribution of HIV-infection in our clinics, more men than women are expected to be enrolled. In this study we use the Reynolds cardiovascular risk score (21) as a vascular covariate in analyses to account for the effect of traditional risk factors associated with vascular disease. We have also elected to enroll patients on stable cART to minimize the effect of uncontrolled viremia.

**Table 1:** Inclusion and exclusion criteria.

**Inclusion criteria**
Seropositive for HIV-1 on the basis of documented HIV infection
On stable cART for at least 3 months prior to screening
Viral load ≤200 copies/mL
Capable of giving informed consent
Aged ≥50 years for *n* = 70 subjects and aged between age 18 and 49 for *n* = 40 subjects
**Exclusion Criteria**
Symptomatic cerebrovascular disease
Uncontrolled diabetes mellitus, hypertension, or familial hypercholesterolemia
Schizophrenia spectrum and other psychotic disorders, untreated Bipolar and related disorders
Chronic seizures, stroke not consistent with CSVD, head trauma resulting in loss of consciousness > 30 min, and multiple sclerosis
Brain infection (except for HIV-1)
Major Neurocognitive Disorder, as established by HAND definition for HIV+ subjects and severity of cognitive and functional impairment for HIV-controls
Serum creatinine levels > 2.0 mg/dl
Current use of immunosuppressants
Chronic inflammatory conditions
Substance use disorder in the past 6 months
Metallic implants that do not meet safety standards for MRI
Claustrophobia

*The inclusion and exclusion criteria were selected to minimize other possible confounding factors. HIV negative controls meet the same inclusion and exclusion criteria except for having a confirmed HIV negative test*.

**Figure 1:**
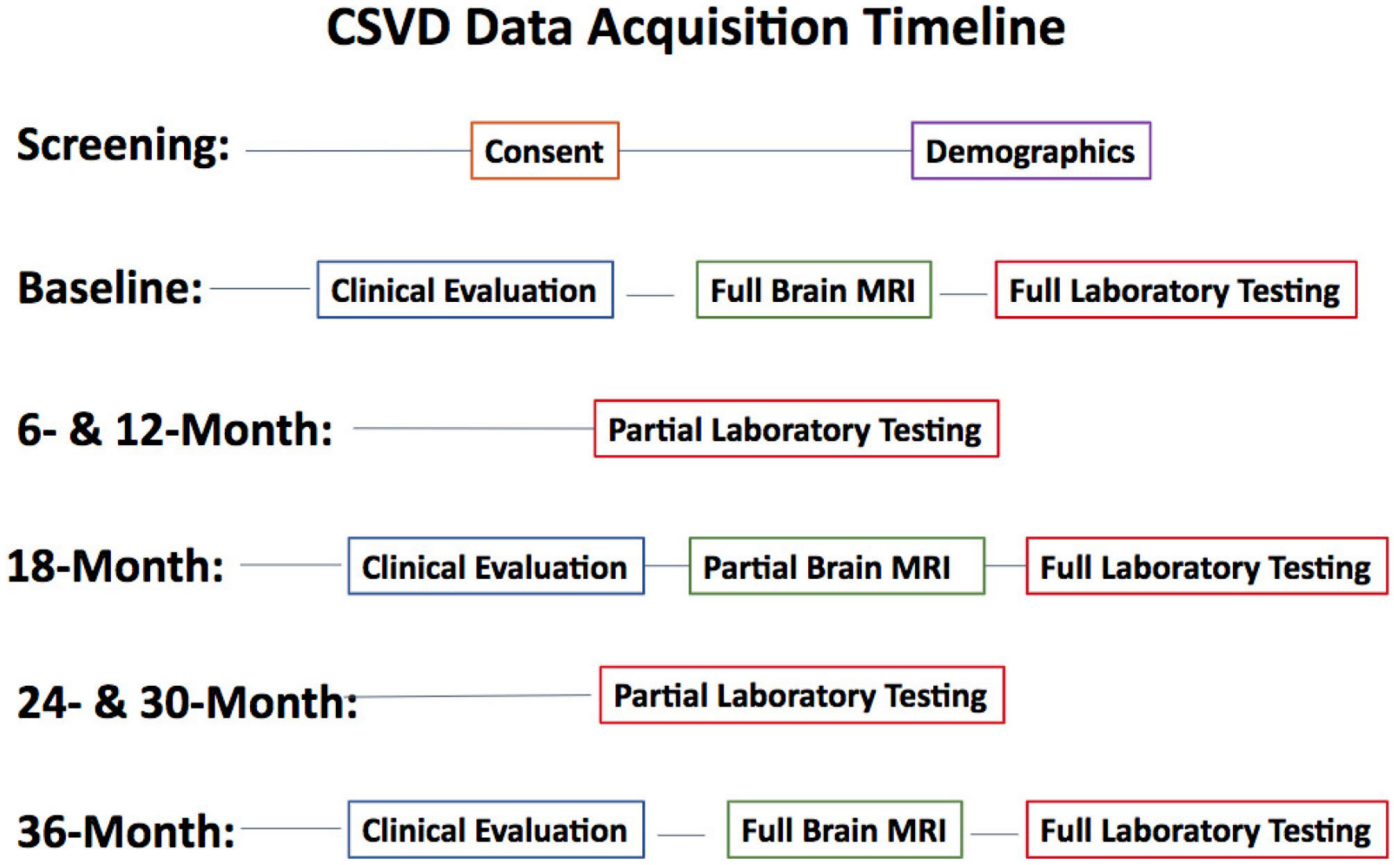
Study data acquisition timeline. Clinical Evaluation includes a physical exam, neuropsychological tests, CES-D, and functional assessments. Full Brain MRI includes all described imaging sequences. Partial Brain MRI excludes QSM, T2w, and MRA sequences. Full laboratory testing includes chemistry, hematology, urine, CD4 count, HIV RNA, urine toxicology, real-time flowcytometry, plasma, and PBMC storage. Partial laboratory testing only includes tests performed for routine clinical care.

Subjects undergo three comprehensive evaluations [baseline (BSL), 18-month, and 36-month time points] that includes clinical evaluation, MRI, and comprehensive blood tests. Routine phone follow-ups are conducted every 6-months after the baseline visit. All collected data is stored in a database in the University of Rochester Medical Center's (URMC) Bio-Lab Informatics System (BLIS), a comprehensive, web-based data management system developed to store, integrate, analyze, and securely share biomedical research data. Details about each type of data collection and processing is described below. See Figure 1 for the study's data acquisition timeline.

### 2.2. Blood Collection and Processing

Approximately 40 ml of whole blood will be collected in Acid Citrate Dextrose (ACD) vacutainers (4 vacutainers) and processed within 2 h of collection. The blood is incubated at room temperature with slow shaking until processing. Plasma is isolated from 20 ml of blood by centrifugation at 1,000 X g for 10 min at room temperature. Plasma is aliquoted and cryopreserved at 80°C for future use. Plasma is used to measure soluble markers of monocyte activation (CD613,HMGB1, and neopterin) as well as endothelial dysfunction (ICAM-1, sVCAM-1, osteoprotegerin, and Lp-PLA2) using commercially available ELISAs. One milliliter of whole blood is processed by flow cytometry to measure the levels of circulating platelet-monocyte complexes (PMCs) and expression of various monocyte activation markers such as c-c chemokine receptor 2 (CCR2), CD40, CD62p (also known as p-selectin), p-selectin glycoprotein ligand (PSGL-1), and tumor necrosis factor receptor 2 (TNFR2). Remaining whole blood is cryopreserved by mixing with RPMI containing 10% Dimethyl sulfoxide (DMSO) and 20% fetal bovine serum (FBS) in 1:1 volume using Mr. Frosty. Cryopreserved whole blood is stored in vapor phase of liquid Nitrogen freezer for future use.

**Figure 2:**
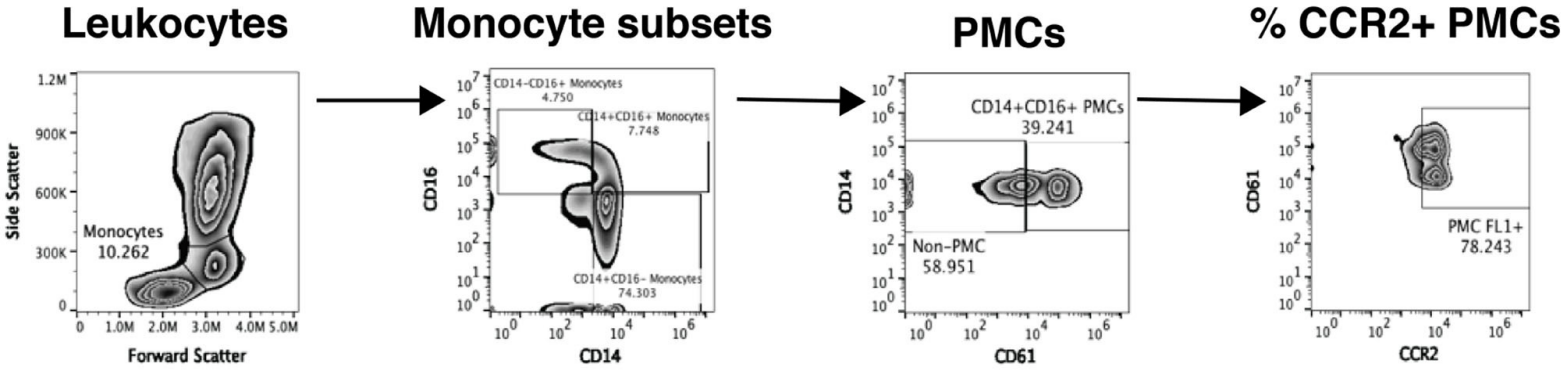
Analysis of platelet-monocyte complexes by flow cytometry. Our flow cytometry gating strategy to analyze PMCs from one representative study sample is shown. Monocytes are initially gated based on forward and side scatter and then split into three subsets based on the expression of CD14 and CD16. Each monocyte subset is then analyzed for expression of CD61, a platelet marker and CD61+ cells are defined as PMCs. Complexed and non-complexed cells are then analyzed for the expression of different monocyte activation markers, namely CCR2 (depicted in the figure), CD40, CD62p, PSGL-1, TNFR2, and TF.

Flow cytometric analysis of platelet-monocyte complexes and platelet activation is shown in Figure 2 and performed as previously described (13, 22). Briefly, 100 μl whole blood is fixed with equal volume of 4% paraformaldehyde (PFA) for 15 min at room temperature followed by red blood cell (RBC) lysis using ACK lysis buffer. The cells are then washed and stained with titrated amounts of antibodies against anti-CD14 PE (BD Biosciences # 555398; 10 μl), anti-CD16 PE Cy7 (BD Biosciences 557744; 3 μl), anti-CD61AF647 (Biolegend # 336408; 3 μl), anti-PSGL-1 FITC (R&D Systems # FAB9961G; 1.5 μl), anti-CD40 FITC BD Biosciences. # 555598; 10 μl), anti-CCR2 FITC (R&D Systems # FAB151G; 1.5 μl), anti-CD62P FITC (BD Biosciences # 555523; 5 μl), and anti-TNFR2 FITC (Miltenyi Biotech #130-107-743; 1 μl) for 30 min at room temperature in the dark. The cells are washed and acquired using the Accuri C6 flow cytometer. 75,000 gated leukocytes are acquired based on forward and side scatter per tube. Data is analyzed using Flow Jo software version 10.4.2. Unstained cells and cells stained with anti-CD14 and anti-CD16 are used to gate on three subsets of monocytes: classical monocytes (CD14+ CD16-), intermediate monocytes (CD14+CD16+), and non-classical monocytes (CD14- CD16+). Among these cells, those that expressed CD61, a platelet marker, are termed as PMCs. Expression of CCR2, CD40, CD62p, and TNFR2 is measured on PMCs and non-complexed monocytes. Further, 10,000 platelet events are acquired based on size beads (0.9–3 μm) and expression of CD61. Platelet activation is measured by expression of CD62p.

### 2.3. Neurocognitive Testing

Assessments of neurocognitive and functional performance are performed in order to derive an HIV-associated neurocognitive disorder (HAND) classification (23). Prior to analyses, Z-scores for each cognitive domain as well as a total Z-score is calculated for each subject. All neuropsychological testing is administered by the study coordinators, trained and supervised by an experienced neuropsychologist. The neuropsychological test battery includes tests of the following cognitive domains: Attention/Working Memory (CalCAP CRT 4; CRT 14), Speed of Information Processing (Stroop Color Naming, Digit Symbol Modalities Test), Executive Function (Trail Making Test B, Stroop Interference Task), Language (letter and category fluency), Learning (Rey Auditory Verbal Learning Test Trials 1–5, Rey Complex Figure Test Immediate Recall), Memory (RAVLT Trial 7, RCFT Delayed Recall), and Motor Skill (Grooved Pegboard). Premorbid intellectual functioning and English language fluency are assessed with the Wide Range Achievement Test (WRAT) 4-Reading subtest at the baseline only.

Functional and mood assessments include the instrumental activities of daily living scale (IADLs) and some activities of daily living (ADLs), self-reported cognitive function via the patient's assessment of own functional inventory (PAOFI), and a measure of depression via the center for epidemiologic studies depression scale (CESD).

### 2.4. MRI Acquisition

All imaging is performed on a research dedicated 3T Siemens MAGNETOM PrismaFit (Erlangen, Germany) whole-body scanner (software version VE11c) equipped with a 64-channel phased array head coil capable of parallel imaging using sensitivity encoding (SENSE), generalized auto calibrating partial parallel acquisition (GRAPPA), and simultaneous multi-slice (SMS) acceleration for diffusion and functional imaging. The maximum gradient strength is 80 mT/m with a slew rate of 200 mT/m/s. Table 2 summarizes the image acquisition parameters used in this protocol.

**Table 2:** Image acquisition parameters.

**Sequence**	**Type**	**TR (ms)**	**TE (ms)**	**FlA (^**°**^)**	**SA**	**Res (mm^3^)**	**TA**
T1w	MPRAGE	1,840	2.45	8	2	1.0 × 1.0 × 1.0	4 m 16 s
T2w	2D	6,000	100	150	1	0.5 × 0.5 × 5.0	2 m 54 s
FLAIR	3D	5,000	215		2	1.0 × 1.0 × 1.0	5 m 40 s
MRA Neck	TOF	20	3.11	20	2	0.6 × 0.6 × 0.8	4 m 22 s
MRA CoW	TOF	21	3.42	20	2	0.5 × 0.5 × 0.5	8 m 37 s
dMRI	2D SE-EPI	4,300	69		3	1.5 × 1.5 × 1.5	11 m 38 s
QSM	3D mGRE	48	5.43	20	2	0.94 × 0.94 × 2.0	7 m 7 s
fMRI	2D GE-EPI	993	43	70	8	2.0 × 2.0 × 2.0	5 m 9 s
pcASL	2D SE-EPI	3,594	19	90	6	2.5 × 2.5 × 2.3	5 m 30 s

*TR, repetition time; TE, echo time; FlA, flip angle; SA, slice acceleration factor; Res, resolution; TA, acquisition time. Sequence specific parameters are located in section 2.5. Total Acquisition time is 55 min 13 s, not including localizers, scouts, and shims, resulting in a total scanner time of about 58 min for baseline and 36-month scans. The 18-month MRI is 32 min 13 s of scanning*.

#### 2.4.1. Clinical Imaging

High-resolution T1w anatomical images are acquired using the magnetization prepared 3D rapid gradient echo (MPRAGE) sequence with the following parameters: inversion time (TI), 926 ms; flip angle (FlA), 8 degrees; echo time (TE), 2.45 ms; receiver bandwidth (RBW), 190 Hz per pixel; echo spacing (ESP), 7.5 ms; repetition time (TR), 1840.0 ms; and 1 mm isotropic resolution. Slices are collected in straight-sagittal orientation. GRAPPA is used with acceleration factor of 2 and 24 reference lines for an acquisition time (TA) of 4 min 26 s.

T2w images are collected in 2D axial orientation with the following parameters: TE, 100 ms; ESP, 11.1 ms; FlA, 150 degrees; RBW, 222 Hz per pixel; TR, 6,000 ms; 0.5 × 0.5 mm in plane resolution and 5.0 mm slice thickness for a TA of 2 min 54 s.

3D FLAIR images are acquired for both clinical readings and quantitative structural processing to improve lesion detection and tissue segmentation on individuals with periventricular atrophy. Imaging parameters include TE, 215 ms; ESP, 3.42 ms; echo train duration, 687 ms; TI, 1,800 ms; RBW, 7,571 Hz per pixel; turbo factor, 278; and TR, 5,000 ms. A slice acceleration factor of 2 is used at 1 mm isotropic voxel size for a total TA of 5 min 40 s.

MRA is acquired using two 3D time of flight (TOF) sequences after a vessel neck scout, centered on the neck and Circle of Willis. Slices are collected in transverse orientation for both scans with flow compensation. GRAPPA is used with acceleration factor of 2 and 24 reference lines. Imaging parameters for the neck/CoW acquisition include TE, 3.11/3.42 ms; FlA, 20/20 degrees; RBW, 252/250 Hz per pixel; TR, 20.0/21.0 ms; phase oversampling, 50/30 %, slice oversampling, 16.7/20.0 %; image resolution, 0.6 × 0.6 × 0.8/0.5 × 0.5 × 0.5 mm^3^; and TA 4 min 22 s/8 min 37 s.

#### 2.4.2. Quantitative Imaging

In addition to the previously mentioned clinical sequences, the diffusion and QSM images described below are also used both for radiological viewing to determine CSVD burden and calculate quantitative metrics via novel image post-processing.

Diffusion imaging is performed using a 2D transverse single-shot single-echo spin echo (SE) echo-planar imaging (EPI) sequence with TE, 69.0 ms; ESP, 0.66 ms; RBW, 1816 Hz per pixel; matrix, 172 × 172; EPI factor, 172; TR, 4300 ms; and 1.5 mm isotropic resolution. Diffusion gradients are applied along 64 directions with two non-zero b-values (1,000 and 2,000 s/mm^2^) and 7 interleaved reference scans. Parallel imaging is enabled using slice acceleration of 3 and phase acceleration of 2 with 40 reference lines for a total scan time of 11 min 38 s. All directions are collected with anterior-posterior (AP) phase encoding. Reference reverse phase encoding b = 0 images are also collected for distortion correction.

QSM is acquired with a 3D multi-gradient echo (mGRE) pulse sequence with the following parameters: 8-echo mono-polar readout train; FlA, 20 degrees; TE of first echo, 5.43 ms; ESP, 1.50 ms; RBW, 930 Hz per pixel; and TR, 48.0 ms. Matrix size is 256 × 256 × 64 with slices in straight-axial orientation and voxel resolution of 0.94 × 0.94 × 2 mm^3^. GRAPPA is used with acceleration factor of 2 and 24 reference lines, giving a scan time of 7 min 7 s.

Blood oxygen level dependent (BOLD) rs-fMRI is performed using a 2D transverse single-shot GE-EPI sequence with TE, 43.0 ms; ESP, 0.56 ms; FlA, 70 degrees; RBW, 2,442 Hz per pixel; EPI factor, 128; TR, 993 ms; and 2 mm isotropic voxels. Parallel imaging is enabled using a multiband acceleration factor of 8 with 12 reference lines for 300 volumes for a total scan time of 5 min 9 s. Images are collected with AP phase encoding. Gradient field maps are also acquired to account for distortion correction. In addition to the rs-fMRI acquisition, we also record three physiological signals for quantitative signal regression, namely heart rate, respiration rate, and end-tidal carbon dioxide (ET-CO2).

Perfusion images are acquired with a multiple post-label delay pseudo-continuous arterial spin labeling (pcASL) sequence using a 2D transverse single-shot single-echo (SE) echo-planar imaging (EPI) sequence with the following parameters: TE, 19.0 ms; ESP, 0.57 ms; FlA, 90 degrees; RBW, 2,326 Hz per pixel; EPI factor, 86; TR, 3594 ms; voxel size 2.5 × 2.5 × 2.3 mm^3^; slice acceleration, 6; and labeling duration, 1.5 s. Multiple tag-control pair measurements are acquired at five delay times for a total of 86 perfusion measurements. One noise pair measurement and one equilibrium pair measurement are also acquired for perfusion calibration processing. The total scan time for these 90 measurements is 5 min 30 s.

### 2.5. MRI Quality Control and Clinical Review

All images are checked by a lab member for quality control within two days of image acquisition in the event that a subject needs to be rescanned due to poor data quality. Quality control consists of confirming proper data transfer from the scanner to the data server, converting the standard digital imaging and communications in medicine (DICOM) files to the neuroimaging informatics technology initiative (NifTI) data format via the dcm2niix tool (24), and visual inspection of all imaging sequences for any scanner and subject related artifacts. Sequences with significant artifacts or excessive subject motion (at least 2 mm shift during a single sequence) are either rescanned at a later date within 30 days of the scan or removed from analyses. In rare cases, sequences may be rescanned during the overall acquisition, time permitting.

Clinical images that are used for diagnostic purposes are also transferred to the Philips IntelliSpace Portal (v10.1) medical software for the team's radiologist to review to determine CSVD severity in each subject and calculate vessel diameters. Radiological findings are immediately documented in the study database in BLIS. Any incidental findings are reported to the principal investigator for review. Documented radiological findings include a Fazekas score (25) rating based on the severity of WMHs, presence of cerebral microbleeds, presence of lacunar infarcts, and enlarged perivascular spaces. We also report measurements of vessel diameters of extracranial cerebral vessels and intracranial vessels forming the Circle of Willis and the basilar artery.

Results of post-processing imaging metrics are also uploaded to the BLIS database, along with all information used for any analyses related to this study. Image postprocessing and obtainable metrics are described in section 2.6. Technical details related to MRI acquisition for each pulse sequence used in our study are described below. Total scanning time is 58 min per subject for each visit.

### 2.6. MRI Data Processing

In this section we describe our comprehensive image analysis pipeline, including preprocessing, data cleaning, and post-processing methods for each imaging modality up to more detailed analyses. Any study specific analyses will be described in future manuscripts dedicated to research aims for this project. We freely provide our image processing pipeline on github to be used for similar studies. All software packages referenced throughout this section can be obtained with proper research credentials and/or collaboration with other laboratories.

**Figure 3:**
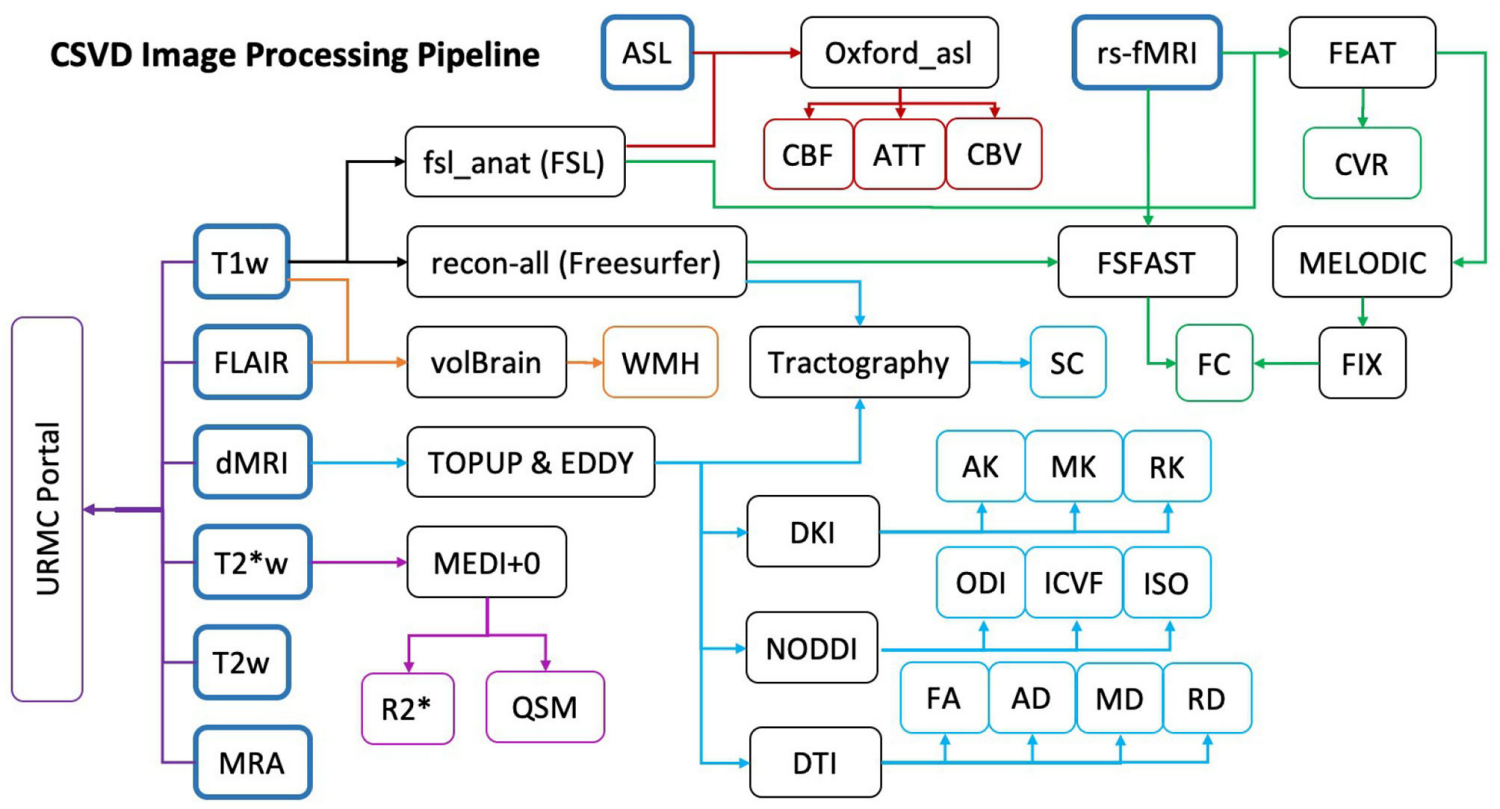
Study MRI data processing pipeline. Colored arrows indicate the steps taken for sequence specific processing: ASL (red), fMRI (green), T1w (Black), FLAIR (Orange), dMRI (Light Blue), T2*w (Purple). Final quantitative metrics calculated from each processing pipeline are indicated in the colored endpoints. The Dark Purple URMC Portal shows the sequences sent to our radiologist for clinical evaluation.

All image processing is completed within the URMC servers either on laboratory desktops or in the Center for Integrated Research Computing (CIRC), except for WMH lesion segmentation. All software used for processing are installed according to proper research agreements. Figure 3 shows our CSVD image processing pipeline. Any deviations from this comprehensive image processing pipeline will be fully detailed in subsequent papers directly pertaining to specific analyses posed.

#### 2.6.1. Structural Imaging

From the structural images collected, we process only the T1w and FLAIR data. In addition to the processing steps described below, we anonymize the T1w NifTI image by removing the face via Freesurfer's mri_deface tool (26). This anonymization ensures that we do not violate any data sharing protocols in certain stages of our processing pipeline. The T2w and MRA images are used exclusively by our team's radiologist for diagnostic purposes. As previously mentioned, all diagnostic findings are maintained in our BLIS database. It is worth noting that there are a few quantitative values calculated from the MRA images. The right and left internal carotid and basilar arterial diameters are measured inside Philips IntelliSpace Portal by our radiologist and uploaded to our database.

##### 2.6.1.1. T1w

Structural segmentation is performed on the T1w images using the anatomical processing script (fsl_anat), available as part of FMRIB's Software Library (FSL) (27–29). Unless otherwise specified, all processing is performed using version 6.0.0. The processing pipeline includes image reorientation and cropping, radio-frequency bias-field correction, linear, and nonlinear registration to Montreal Neurological Institute (MNI)-152 2 mm standard space via FLIRT (30, 31) and FNIRT (32) respectively, brain extraction via BET (33), tissue segmentation with FAST (FMRIB's Automated Segmentation Tool) (34), and subcortical structure segmentation using the FIRST algorithm (35).

Cortical reconstruction and volumetric segmentation is performed with the Freesurfer image analysis suite, which is documented and freely available for download online (http://surfer.nmr.mgh.harvard.edu/). The technical details of these procedures are described in prior publications. Briefly, this processing includes motion correction and averaging (36) of multiple volumetric T1w images (when more than one is available), removal of non-brain tissue using a hybrid watershed/surface deformation procedure (37), automated Talairach transformation, segmentation of the subcortical white matter and deep gray matter volumetric structures (including hippocampus, amygdala, caudate, putamen, ventricles) (38, 39) intensity normalization (40), tessellation of the gray matter white matter boundary, automated topology correction (41, 42), and surface deformation following intensity gradients to optimally place the GM/WM and GM/CSF borders at the location where the greatest shift in intensity defines the transition to the other tissue class (43, 44). Once the cortical models are complete, a number of deformable procedures can be performed for further data processing and analysis including surface inflation (45), registration to a spherical atlas which is based on individual cortical folding patterns to match cortical geometry across subjects (46), parcellation of the cerebral cortex into units with respect to gyral and sulcal structure (39, 47), and creation of a variety of surface based data including maps of curvature and sulcal depth. This method uses both intensity and continuity information from the entire three dimensional MR volume in segmentation and deformation procedures to produce representations of cortical thickness, calculated as the closest distance from the GM/WM boundary to the gray/CSF boundary at each vertex on the tessellated surface (44). The maps are created using spatial intensity gradients across tissue classes and are therefore not simply reliant on absolute signal intensity. The maps produced are not restricted to the voxel resolution of the original data thus are capable of detecting submillimeter differences between groups. Procedures for the measurement of cortical thickness have been validated against histological analysis (48) and manual measurements (49, 50). Freesurfer morphometric procedures have been demonstrated to show good test-retest reliability across scanner manufacturers and across field strengths (51, 52).

##### 2.6.1.2. FLAIR

We use both the anonymized T1w and the FLAIR images to quantitatively determine total WMH lesion burden. To do so we use volBrain, an automated online MRI brain volumetry system (53). More specifically, we make use of the lesionBrain pipeline to segment and classify any WMH lesions in our subjects (54). The lesionBrain pipeline consists of the following stages: preprocessing, structure segmentation, candidate mapping, lesion segmentation, and lesion classification. Upon completion of the pipeline, a detailed report is generated and includes the overall lesion load, the number of lesions in each lesion class (periventricular, juxtacortical, deep white, and infratenorial). We use this quantitative measure of WMH burden to supplement, rather than corroborate, our clinical findings for future analyses.

Briefly, preprocessing of the images includes intensity normalization and registration to MNI space, denoizing by an adaptive nonlocal means filter (55), correction for inhomogeneity (56, 57), and tissue segmentation [gray matter (GM), white matter (WM), and cerebrospinal fluid (CSF)] (58). Structure segmentation further segments the following brain structures: intracranial cavity (ICC), brainstem, cerebellum, and lateral ventricles (59–61). Candidate mapping is performed only in areas where lesions are likely to be found. The mean (μ) and standard deviation (σ) of the GM FLAIR intensities are used to estimate a threshold (thr = μ + ασ, where α = 0.5) above which all voxels within the ICC mask are considered as lesion candidates. Due to some inconsistencies in training the algorithm compared to the lesionBrain training dataset, any voxels that lie within a previously built lesion atlas are also considered as lesion candidates (62, 63). Lesion segmentation is performed using an extension of the RI-NLM method (64). Segmentation is performed in two stages to account for challenges associated with both a voxel-based and patch-based approach. The RI-NLM method is first applied to the T1w and FLAIR images to obtain a lesion probability mask. This is followed by a secondary regularization step using a patch-wise denoizing filter (65). The weights of the filter are estimated on the FLAIR and used to average the lesion probabilities. Systematic error correction is automatically used to produce the final lesion segmentations (66, 67). Finally, a classification algorithm is used to classify lesion type.

#### 2.6.2. Diffusion Imaging

With a high angular resolution diffusion imaging (HARDI) acquisition, we are able to derive many quantitative metrics using various post-processing techniques. This section covers the following stages of our diffusion image processing pipeline: preprocessing, diffusion tensor imaging (DTI), diffusion kurtosis imaging (DKI), neurite orientation dispersion and density imaging (NODDI), GM and WM based spatial statistics (GSBSS and TBSS), tractography, and structural connectivity (SC) extraction.

The preprocessing pipeline for all subsequent post-processing included brain extraction using FSL's BET (33), susceptibility induced distortion correction using TOPUP (28, 68), and eddy-current induced distortion and subject motion corrections using EDDY (69). Data is collected with reversed phase-encode blips, resulting in pairs of images with distortions going in opposite directions. From these pairs the susceptibility-induced off-resonance field is estimated using a method similar to that described in (68) as implemented in FSL and the two images are combined into a single corrected one. More technical information about FSL's EDDY can be found in (68–70). This preprocessing pipeline is applied to all subsequent post-processing methods, described below. Images are manually inspected using the motion correction reports and data visualization software upon completion of data preprocessing to catch any errors during preprocessing. Errors during preprocessing are corrected on an individual basis, as needed.

Whole-brain voxel-wise metrics are calculated from various post-processing techniques to provide more information about the diffusivity properties of brain tissues. We obtain ten different metrics from our HARDI data that is modeled by three theoretical diffusion signals. Diffusion tensor imaging (DTI) is a model fitting technique that fits the diffusion tensor model (71). Metrics are fit to each voxel using a linear regression with sum of least-squares error and fractional anisotropy (FA), axial diffusivity (AD), mean diffusivity (MD), and radial diffusivity (RD) maps using FSL's DTIFIT. Diffusion kurtosis imaging (DKI) extends the second order diffusion tenor model to a fourth order kurtosis model to fit additional metrics (72). Axial kurtosis (AK), mean kurtosis (MK), and radial kurtosis (RK) metrics are calculated using the DKI estimator in the Diffusion Imaging in Python (DIPY) module in Python 3.6 (73). The DKI estimator in DIPY automatically fits the four tensor metrics as well. As part of quality control checks of the processed data, we correlate the tensor metrics fit by DTI and DKI to ensure within-subject consistency prior to analyses. Finally, neurite orientation dispersion and density imaging (NODDI) estimation is processed using the NODDI Toolbox v1.0.1 run on the MATLAB platform (R2018a). NODDI estimation fits each parameter of the NODDI model using maximum likelihood estimation using a Rician noise model with the Gauss-Newton nonlinear optimization technique (74). The voxel-wise metrics produced from the NODDI model are the neurite density or intracellular volume fraction (ICVF), orientation dispersion index (ODI), and extracellular or isotropic volume fraction (ISO). These NODDI parameters are reliable in the GM in addition to WM. In summary, we fit ten quantitative diffusion metrics from three different signal models using our HARDI data.

Since we build so many whole-brain voxel-wise metrics, it is important that we prepare each map for group-based analyses. On a voxel-wise scale, the most common group analysis is whole-brain spatial statistics. While we do not present specific spatial statistical analyses, we do present two options for spatial statistics and describe the steps taken to prepare for each. The main option we use for group analysis is tract-based spatial statistics (TBSS) in FSL (28, 33, 75–77). In order to perform TBSS along WM tracts, three main steps are required. The first step is nonlinear alignment of each subject's FA map to the FMRIB58_FA 1 mm standard space target via FNIRT. Second, a skeleton projection is created by thresholding the FA intensities of all subjects. Finally, voxel-wise statistics is performed on the skeleton using FSL's randomize with research specific hypothesis testing (78). Due to the high quality HARDI and rs-fMRI acquisitions that we use, we have the option of also performing NODDI-improved GM-based spatial statistics (N-GSBSS) using the N-GSBSS pipeline (79). The N-GSBSS pipeline is self-contained and does not directly follow from any preprocessing described above.

The final stage of diffusion processing is tractography in order to calculate SC matrices for each subject. After diffusion preprocessing, the eddy corrected diffusion weighted images are postprocessed using MRtrix3 (www.mrtrix.org), Advanced Normalization Tools (ANTs) (80), and the Sherbrooke Connectivity Imaging Lab toolbox in python (Scilpy) as part of the population-based SC (PSC) pipeline (81). The b0 reference image is extracted, skull stripped, bias-field corrected, cropped, intensity normalized, and resampled to 1 mm isotropic resolution. The high-resolution T1w anatomical image is then registered to the high-resolution diffusion image using ANTs registration tools before being passed through Freesurfer's recon-all pipeline. Once the standard atlas parcellations are rigidly registered to this diffusion anatomical space, the fiber orientation distribution function (fODF) is calculated within each voxel to prepare for tractography (82). Tractography is performed using a particle filtering tractography (PFT) algorithm (83). Finally, invalid streamlines are removed and SC matrices are calculated for both the Desikan-Killiany (47) and Destrieux (84) atlases using the PSC toolbox.

#### 2.6.3. Quantitative Susceptibility Mapping

QSM reconstruction is performed utilizing the morphology enabled dipole inversion with zero-tissue referencing (MEDI+0) toolbox (85–88), on the MATLAB R2018a environment (The Mathworks, Inc., Natick, MA). Phase unwrapping is performed and background field removal is completed using the projection onto dipole field (PDF) method. R2* maps are produced via a monoexponential curve fitting and used to create CSF masks to use as anatomical prior information before performing MEDI. Finally, the dipole inversion is performed using L1 regularization with Lagrange multipliers λ set to 1,000 and λ_*CSF*_ set to 100, spherical mean value (SMV) radius set to 5, and the model error reduction through iterative tuning (MERIT) option (89). R2* maps are calculated during the QSM reconstruction process and separately saved. Zero-tissue referencing is done with respect to CSF and incorporated into the reconstruction toolbox to allow for more accurate intersubject comparisons.

Both QSM and R2* maps are coregistered to the high resolution structural and MNI152-2 mm standard spaces via the magnitude image of the first echo of the data. Linear registration between the T1w and QSM map is performed using FLIRT, while FNIRT nonlinear registration is used to register the QSM map to standard space. Whole-brain voxel-based comparisons are performed in standard space using FSL's randomize tool (78) with family-wise error rate correction after gaussian smoothing of 5 mm to account for coregistration errors and other susceptibility induced artifacts.

#### 2.6.4. Functional Imaging

Similar to the diffusion processing pipeline, our functional images can be used for more than one purpose. Our functional processing pipeline includes the following steps: volumetric preprocessing, denoizing, and resting state network (RSN) extraction, whole-brain voxel-wise CVR calculation, volumetric functional connectivity (FC) calculation, and surface-based preprocessing, denoizing, RSN extraction, and FC calculation. While others have proposed to incorporate both volumetric and surface-based functional processing data for more integrated information, we keep our volumetric and surface-based processing independent of each other. Steps may be adopted in the future to incorporate both types of functional processing, depending on more specific analyses.

##### 2.6.4.1. Volumetric Functional Processing

FMRI data processing is carried out using FSL's FMRI Expert Analysis Tool (FEAT) Version 6.00. Registration to high resolution structural is carried out using boundary-based registration (BBR) (90). Registration from high resolution structural to standard space is then further refined using FNIRT nonlinear registration (32, 77). The following pre-statistics processing is applied: motion correction using MCFLIRT (91); slice-timing correction using Fourier-space time-series phase-shifting; non-brain removal using BET (33); spatial smoothing using a Gaussian kernel of FWHM 5 mm; grand-mean intensity normalization of the entire 4D dataset by a single multiplicative factor; high-pass temporal filtering (Gaussian-weighted least-squares straight line fitting, with sigma = 50.0 s).

Independent component analysis (ICA) based exploratory data analysis is carried out using probabilistic ICA (92) as implemented in FSL's Multivariate Exploratory Linear Decomposition into Independent Components (MELODIC) Version 3.15 in order to investigate the possible presence of unexpected artifacts or activation. The following data preprocessing is applied to the input data: masking of non-brain voxels; voxel-wise demeaning of the data; and normalization of the voxel-wise variance. Preprocessed data are whitened and projected into a 64-dimensional subspace using probabilistic principal component analysis (PCA) where the number of dimensions is estimated using the Laplace approximation to the Bayesian evidence of the model order (92, 93). The whitened observations are decomposed into sets of vectors which describe signal variation across the temporal domain (time-courses) and across the spatial domain (maps) by optimizing for non-Gaussian spatial source distributions using a fixed-point iteration technique (94). Estimated component maps are divided by the standard deviation of the residual noise and thresholded by fitting a mixture model to the histogram of intensity values (92).

After all functional preprocessing and exploratory ICA analysis has been completed, we use FMRIB's ICA-based Xnoiseifier (FIX) to automatically classify “good” and “bad” components in order to remove the “bad” components from the time series data (95, 96). FIX runs on FSL, MATLAB, and R (97), with a series of dependent packages in each. Due to the similar nature of our functional data to the Human Connectome Project's (HCP), we utilize the pretrained weights from the “minimally-preprocessed” 3T HCP-like datasets (TR = 0.7 s, 2 mm isotropic resolution, 15 min session, minimal high pass temporal filtering) (95, 98). Unfortunately, our scanning parameters are not identical to HCP's, so we do expect some classification errors due to the nature of the FIX algorithm. As such, all images and components are manually checked for consistency before removing “bad” components.

We calculate the CVR index for each voxel in standard and native spaces following the method described in (99). Note that CVR is calculated before MELODIC and FIX processing by in house scripts. CVR is an indirect measure of how blood vessels respond to changes in carbon dioxide. However, due to the resting state nature of our study, we use a data-driven approach to calculate reactivity. Others have previously demonstrated that reactivity signals are part of the average BOLD signal of all GM voxels (99). In order to calculate CVR, we first perform a band pass filter to obtain the 0.02–0.04 Hz frequency range. We then take the average BOLD time course of all GM voxels of an individual and use it as a regressor against the voxel-wise time-course of every voxel in the brain. The regression coefficient associated with this linear model is then normalized by a reference tissue to obtain the relative CVR of every voxel.

Finally, we use the denoized functional data to build FC matrices between regions of the brain. There is no consensus on the best way to calculate FC (100). We use the atlas-based FC Pearson correlation (101) implementation in the Nilearn: Machine learning for Neuro-Imaging in Python module (102). We incorporate the standard nuisance regressors: translational and rotational motion, WM, CSF, and the global signals. We build FC matrices with and without various combinations of nuisance regressors for both the Desikan-Killiany (47) and Destrieux (84) atlases, similar to the SC matrices. By saving matrices with different combinations of regressors, we provide options for many kinds of future analyses without having to recalculate FC matrices.

##### 2.6.4.2. Surface-Based Functional Processing

Surface-based fMRI preprocessing is carried out using the Freesurfer functional analysis streamline (FSFAST) pipeline using Freesurfer version 6.0.1. The following pre-statistics processing is applied: motion correction using MCFLIRT (91); slice-timing correction using Fourier-space time-series phase-shifting (103); non-brain removal and masking; registration to the anatomical; sampling to the surface; and surface smoothing using a Gaussian kernel of FWHM 5 mm. Surface sampling is performed onto the left and right hemispheres in the native anatomical (diffusion) space. Once the functional preprocessing steps are completed, nuisance regressors are calculated using principal component analysis (PCA). Surface-based registration methods in Freesurfer are used to register individual native surfaces to the fsaverage mesh space prior to any group-wise analyses (45). We use surface-based adaptions of all of the volumetric post-processing methods to obtain surface-based RSNs and FC matrices. Additionally, surface-processed rs-fMRI is used to derive seed-based connectivity maps to compare differences between groups with better spatialization on the cortical surface. While it is possible to combine both the volumetric and surface-based fMRI data after preprocessing, as has been done in the HCP minimal preprocessing pipelines (104), we do not utilize the HCP pipeline due to the 2D nature of our T2w images.

#### 2.6.5. Perfusion Imaging

ASL images are processed using the Oxford ASL tool (105–107). Preprocessing includes motion correction using MCFLIRT, slice timing correction, distortion correction, spatial regularization, and registration to high resolution anatomical space using the boundary-based registration (BBR) algorithm (90) and to MNI152-2 mm standard space using FNIRT nonlinear registration. CBF quantification is run in three steps: Bayesian inference for CBF according to the Buxton kinetic curve model (108), Bayesian inference of further parameters as applicable to multi-delay data, including cerebral blood volume (CBV) and arterial transit time (ATT), and Bayesian inference with spatial priors to fine tune the model parameters initialized by the high-resolution anatomical image.

#### 2.6.6. Region of Interest Metric Calculations

For pre-specified regions of interest (ROIs), we also calculate average ROI values for all 16 previously described quantitative metrics: DTI (FA, AD, MD, and RD), DKI (AK, MK, and RK), NODDI (ISO, ICVF, and ODI), QSM (QSM and R2*), fMRI (CVR), and ASL (CBF, ATT, and CBV). We use the following atlases for ROI extraction, available in FSL in standard MNI152-2 mm space: Harvard-Oxford cortical (HO-cor) and subcortical (HO-sub) (47, 109–111) and the Johns Hopkins University WM tracts (JHU-tracts) (112–114). To obtain the most accurate averages for each metric, we take each ROI defined in each atlas and apply nonlinear and linear inverse warps with FNIRT and FLIRT to warp each ROI to native metric spaces. After warping, ROIs are binarized before taking the average metric within each ROI. Each value is uploaded to BLIS for future analyses. The only metric that is not averaged in native space is the CVR due to some theoretical limitations described in the literature (see Discussion).

### 2.7. Statistical Analyses

#### 2.7.1. Sample Size Calculation

The sample size calculation reflects differences in imaging metrics previously reported by our group and others in HIV infected individuals compared to HIV uninfected individuals. Here we present a power analysis starting with DTI metrics derived from one of our studies on patterns of white matter injury in HIV infection (115).

##### 2.7.1.1. Diffusion Tensor Imaging

Fifty HIV participants and 13 HIV-uninfected controls were used in that study. From the results of this study, we selected two brain areas that provide reliable and reproducible DTI measurements, namely the body and splenium of the corpus callosum (BCC and SCC), although we identified other white matter structures that show even more significant differences between HIV- controls and HIV+ subjects. Therefore, the power calculation should err on the conservative side. The mean MD value among controls for the BCC and SCC is 0.964, the pooled standard deviation computed from all subjects is 0.049, and the observed effect size is 0.0371, which represents a 3.8% difference based on the sample mean of the controls. The power is calculated based on the information from this preliminary study using a two-sided *t*-test for comparing two groups, which is reported in Table 3.

**Table 3:** Statistical Power for two-sample Student *T*-test based on mean MD of the BCC and GCC.

	***d* = 2.6%**	***d* = 3.0%**	***d* = 3.4%**	***d* = 3.8%**
*n* = 60	0.794	0.894	0.953	0.982
*n* = 65	0.825	0.916	0.966	0.988
*n* = 70	0.852	0.934	0.976	0.992
*n* = 75	0.875	0.949	0.983	0.995
*n* = 80	0.895	0.960	0.988	0.997
*n* = 85	0.912	0.969	0.991	0.998

*n is the sample size of each group and d is the minimum effect size as the percentage of the control group that can be detected. The significance level is α = 0.05*.

Based on this table, if we assume to have at least 60 HIV- control subjects and at least 60 HIV+ subjects, we can detect a very conservative effect size of 3.0% with about 90% power between the two groups. A similar power calculation can be derived using data from a recent publication from (116) assessing brain white matter hyperintensity in HIV infection. The median (IQR) periventricular WMH load for the HIV+ group (*n* = 103) and control group (*n* = 70) is 0.8(1.5) and 0.4(1.0), respectively. If we recruit 80 subjects in each group, we have 80% of statistical power at significance level α = 0.05. However, in both our previous study and (116) the mean age was around 50 years of age. To further investigate age-CSVD interaction, we will also include subjects younger than 50 years old. We assume that the frequency of CSVD will decrease with the addition of younger subjects but it will still be more frequent in HIV+ compared to HIV- subjects. Therefore, assuming conservatively that the true difference may be only 2.6% (if younger subjects are included) as opposed to around 3.8% (if only older subjects were included), if we have *n* = 85 subjects per group, we can achieve more than 90% statistical power. Furthermore, considering a drop-out rate of about 20%, we plan to recruit *n* = 110 subjects per group for a total of 220 subjects.

##### 2.7.1.2. Cerebral Blood Flow (CBF)

Ances et al. (21) measured resting CBF in the lenticular nuclei of HIV+ (*n* = 33, mean = 47.1) and HIV- individuals (*n* = 26, mean = 56.2). The pooled standard deviation was 8.1. Based on these results, the statistical power is more than 90% even if we only have *n* = 30 in each group.

##### 2.7.1.3. Quantitative Susceptibility Mapping (QSM)

Limited information is available in the literature on HIV-associated changes in brain tissue susceptibility. Miszkiel et al. (117) measured R2* in several ROIs of HIV infected (*n* = 28) and uninfected (*n* = 15) subjects. In the globus pallidus, the mean group differences and pooled standard deviations are 0.0029 and 0.0027, respectively; in the caudate, the group difference is smaller (0.0019) and the pooled standard deviation is larger (0.0037). By using the conservative results from the caudate, we can still achieve 91% statistical power with *n* = 85 subjects in each group.

##### 2.7.1.4. Cognitive Function

Decreased cognitive performance is well documented in HIV infection and demonstrable even with small sample size (118, 119). In our previous studies, we observed a mean Z-score of −1.14 in HIV+ subjects compared to a mean Z-score of 1.46 and a pooled standard deviation of 4.02. Thus, with *n* = 85 in each group, the statistical power is 99%. However, our goal goes beyond a comparison between HIV+ and HIV- subjects. Rather, we will use a high-dimensional multivariate regression analysis with a data-driven model selection procedure and multiple testing adjustment. We are confident that most, if not all, of the multivariate associations investigated will be significant, because for *n* = 85 in each group, we have adequate power for these covariates in the marginal power analyses.

#### 2.7.2. Analysis Plan

Demographic and clinical data are assessed by descriptive analysis using means and standard error (SE), medians and inter-quartile ranges for continuous variables, and proportions for categorical variables. We use graphical methods such as histograms, Q-Q plots, and box-plots to visualize the data and identify potential data problems such as outliers, missing data, and skewness. If problems are detected, appropriate data preparation steps such as outlier removal, data imputation, and log-transformations are considered.

Comparisons between two independent groups (e.g., HIV+ versus HIV- subjects at baseline) are conducted by either two-group Welch's *t*-test or Wilcoxon ranksum test (for continuous variables), or Fisher's exact test (for categorical variables). One-way analysis of variance (ANOVA) *F*-test followed by Tukey's *post-hoc* test and Kruskal-Wallis test with Dunn *post-hoc* test are used to compare continuous variables across 3 or more (*K* ≥ 3) groups. Paired *t*-test and Wilcoxon signed-rank test are used to compare the levels of continuous variables between two visits. Repeated measures ANOVA *F*-test and Friedman test are used to test marginal group differences across multiple time points. Pearson and Spearman correlation analyses are used to assess marginal associations between two continuous variables. The R package *cocor* is used to compare the correlation coefficients computed from the two cohorts (120). A *p*-value < 0.05 is considered statistically significant for a single hypothesis testing problem. For inferential problems that involved multiple hypotheses, the Benjamini-Hochberg multiple testing procedure (121) is used to control the false discovery rate (FDR) at the < 0.05 level. All statistical analyses are performed in R (R Foundation for Statistical Computing, Vienna, Austria).

Multivariate regression models are used to quantify the associations between selected covariates and continuous response variables at BSL, 18-month, and 36-month visits, controlling for potential confounding effects such as age. The response variable and covariates in these regression models depend on specific research aims. For example, at BSL, we can designate cognitive function (measured by the neuropsychological tests) as the response variable and associate it with a host of covariates including brain injury (measured by various brain imaging markers), HIV status (cohort), cART treatment, cardiovascular risk score, and age. As an extension, linear mixed effects regression (LMER) models are be applied to data collected at all three visits from both cohorts. We use per-subject random intercepts to account for serial correlations between multiple time points. If needed, certain interaction terms may be included. For example, we may include the interaction between visit and age to account for potential pattern differences of cART treatment effects between young and old subjects. In these LMER analyses, parameters are estimated by the restricted maximum likelihood (REML) criterion, and the statistical significance is assessed by the adjusted ANOVA *F*-test provided by R package lmerTest (122). For both cross-sectional multivariate regression and longitudinal LMER analyses, the fitted linear coefficients, their 95% confidence intervals, and the corresponding *p*-values are reported.

Since multiple types of data are used in this study and many of them are high-dimensional, it helps to use both domain knowledge and data-driven methods to select compact models and prevent model overfitting. For example, instead of including all ROIs in inferential statistical analyses, we prioritize those ROIs with known relationship with the outcome variables as documented by previous literature or our preliminary studies. Data driven methods, such as factor analysis, cluster analysis, as well as statistical model selection methods for regression can be used to further reduce the number of hypotheses to be tested or covariates in regression models. Step-wise methods based on Akaike Information Criterion (AIC) and/or Bayes Information Criterion (BIC) can be used for models with low to moderate numbers of covariates (*p* ≤ 20). For even larger models, we apply penalized regression techniques such as LASSO (123), elastic net (124), and SCAD (125), instead.

## 3. Discussion

CSVD in HIV infected individuals is likely a multifactorial disease where there is a convergence of traditional vascular risk factors (hypertension, hyperlipidemia, diabetes, and age) and HIV mediated chronic inflammation. The prevalence of CSVD in HIV infected individuals over the age of 50 has been reported with a wide range from 48 to 80% (7, 116). A recent study which enrolled 456 HIV+ and 154 HIV- subjects found that 51.5% of HIV+ vs. 36.4% of HIV- subjects had signs of CSVD (126). HIV infection in this study is considered as an independent risk factor for CSVD. CSVD and HIV infection may also have a synergistic effect on brain function and increase the risk of cognitive impairment in this vulnerable population. CSVD is associated with an increased likelihood of HAND (10), though there is some evidence that the contribution of CSVD to cognitive impairment is independent of that of HIV infection itself (127). In the cART era, individuals living with HIV infection have significantly increased life expectancy; however, quality of life may be reduced with significant rates of HAND. CSVD may be a modifiable risk factor of disease morbidity; identifying reliable surrogate biomarkers would be essential for targeting interventions.

### 3.1. Blood Biomarkers

Two recent reports have linked soluble markers released by monocytes and endothelial cells with CSVD in the general population, including neopterin, sICAM-1, sVCAM-1, osteoprotegerin, and Lp-PLA2 mass (11, 12). It is interesting that neopterin, typically produced by activated monocytes/macrophages, is also a marker of HIV-associated CNS injury (128). Furthermore, monocyte activation is associated with decreased cognitive function in HIV+ individuals (129) and there is preliminary evidence that reductions in monocyte activation following treatment with a C-C chemokine receptor type 5 (CCR5) antagonist are associated with improvements in cognitive function, particularly in the domains of attention and working memory (130).

Lp-PLA2 is also secreted by myeloid cells such as macrophages and is involved in cleavage of the oxidized phosphatidyl-choline component of LDL particles and thus has been strongly associated with atherosclerosis (131). Two additional markers derived from monocytes have been implicated in atherosclerosis and are elevated in HIV infection: sCD163 and HMGB1 (132–136). CD163, a monocyte-specific scavenger receptor, is shed during activation as soluble CD163 (sCD163) in HIV-infected individuals prior to and after cART treatment (137). HIV+ patients exhibit elevated levels of sCD163 and neopterin in an age-dependent manner (138–140), which is even more concerning for atherosclerosis in older HIV+ individuals.

HMGB1 is a nuclear factor and a secreted protein (141). HMGB1 is secreted by activated MΦ (142) and also rapidly leaked when membrane integrity is lost in permeabilized or necrotic cells (143). Extracellular HMGB1 and its receptors, RAGE, TLR2, and TLR4, have been implicated in mechanisms of many inflammatory diseases, including sepsis, atherosclerosis, and rheumatoid arthritis (144) by induction of senescence-associated secretory phenotype via NF-kB activation (145). Plasma levels of HMGB1 are elevated during the course of HIV infection and possibly associated with high viral load (135).

Platelets also play a major role in very early stages of response to an injury by trauma or infection. Platelets are very sensitive to inflammatory stimuli and are increasingly recognized as important immune mediators. Increased platelet activation is widely observed in patients with HIV infection (13, 146–148). Their high numbers in circulation compared with that of circulating leukocytes and ability to release pro/anti-inflammatory mediators stored in secretory granules suggest that platelets are critical players in the early phase of the host immune response (149–152). Platelets regulate functions beyond homeostasis and have been identified as a functional player in both innate and adaptive immune systems (153, 154). In the immune system, one of these functions is to promote trafficking of immune cells into injured tissues by upregulation of P-selectin (CD62P) and CX3CR1, leading to enhanced rolling of leukocytes along the vascular endothelium. Thus, activated platelets mediate the interaction between the endothelium and circulating immune cells and can participate in the pathology of the disease during acute and chronic inflammatory conditions (155–158).

### 3.2. Magnetic Resonance Imaging

Beyond the standard clinical MRI sequences used to diagnose CSVD in our study participants, we use a novel combination of advanced MRI sequences to explore the underlying pathomechanisms of HIV-associated CSVD. Several studies have documented HIV-associated central nervous system (CNS) injury and specifically white matter microstructure changes (115, 159, 160), but these findings have not been evaluated in the context of CSVD. Given the aging HIV population, the prevalence of CSVD is going to increase, contributing to altered brain connectivity and cognitive impairment (161, 162).

In order to interrogate these WM microstructural changes, we use a HARDI sequence that allows the reconstruction of quantitative microstructural metrics throughout the brain. Studies have shown that in aging populations, FA decreases while AD, MD, and RD increase (163). Similarly, NODDI metrics have also shown to incur significant changes in older adults compared to controls; ODI increases while ICVF and ISO tend to decrease with aging (164). Given our rs-fMRI sequence, we are able to obtain individual and group profiles of resting state networks (RSNs) and CVR. Based on previous studies, we expect RSNs to deviate in a cohort with chronic inflammatory processes compared to controls (165).

The Human Connectome Project (HCP) is considered the current gold standard for connectivity data and analyses (98). We have taken advantage and adopted many of the recommendations of the HCP, building a robust processing pipeline that will be sensitive to SC and FC patterns thus reflective of cognitive changes. For example, we are able use our diffusion sequence to perform high quality streamline tracking to estimate the WM fiber curves of our participants, allowing us to build more reliable SC profiles than standard clinical diffusion sequences. Group effects due to chronic inflammatory processes have shown changes in SC that correlate with cognitive measures (166). Additionally, we collect rs-fMRI with a low TR and high spatial resolution, which gives us the flexibility to perform both volumetric and surface-based image processing and analyses. As such, we can build FC profiles for each participant, which have also been shown to deviate from controls in the presence of chronic inflammation and CSVD (161).

CVR via rs-fMRI is reflective of the ability of cerebral blood vessels to respond to vasoactive stimuli, which has been shown to correlate with cognitive decline in older adults and groups impacted by vascular diseases (167). The approach we use to measure CVR does not provide absolute quantification at the voxel level. However, a recent study has demonstrated that it may be possible to adjust relative CVR values by tissue referencing across a population (168).

With the presence of WMHs, subcortical iron deposition concentration has been shown to increase, likely due to oxidative stress on the brain (169). QSM is sensitive to changes in iron deposition and is likely useful to assess longitudinal changes in tissue susceptibility concentration (88). Additionally, R2* mapping is sensitive to tissue differences in tissue susceptibility distribution. Incorporating measures from both QSM and R2* mapping has the potential to assess iron related changes in the brain, especially in the deep gray matter with high vasculature (170).

Finally, perfusion metrics have been shown to be direct measures of vascular health. Decreased regional CBF is seen in the early stages of HIV infection (171) and has been associated with vascular risk factors such as increased triglyceride levels in HIV+ men (172). Changes in arterial thickness and extra-cranial vessel diameters have been shown in the context of HIV (173). By incorporating measures of larger intra- and extra-cranial vessel diameters (by MRA), we are able to assess the extent to which HIV impacts blood flow in the context of CSVD. This study allows us to relate the blood markers of vascular disease to the presence of cognitive impairment, identify regional areas of hypoperfusion, and determine how they relate to specific patterns of cognitive dysfunction.

## 4. Conclusion

This study protocol description serves to outline a comprehensive longitudinal study at the University of Rochester to study the effects of aging and HIV on CSVD and provide detailed data acquisition parameters for others to implement similar neuroimaging studies at or higher than the current standard for neuroimaging studies about CSVD without contrast agents. We also provide a comprehensive data processing pipeline, available on github, and detail every step taken for future analyses. Specific aims associated with this study will be addressed in a series of baseline and longitudinal follow-up analyses upon successful collection and processing of all data necessary for each aim.

## Ethics Statement

The studies involving human participants were reviewed and approved by University of Rochester Research Subjects Review Board. The patients/participants provided their written informed consent to participate in this study.

## Author Contributions

KM drafting/revising of the manuscript for content, image processing pipeline creation, and acquisition and interpretation of data. MU, YZ, MT, MS, XQ, HW, MW, and JZ revising manuscript for content and acquisition and interpretation of data. SM and GS revising of the manuscript for content, including medical writing, study concept, design, and acquisition and interpretation of data. All authors have read and approved the manuscript before submission.

## Conflict of Interest

The authors declare that the research was conducted in the absence of any commercial or financial relationships that could be construed as a potential conflict of interest.
